# Influence of Prednisolone Treatment on Serum Bile Acid Concentrations in Cats

**DOI:** 10.3390/vetsci12100933

**Published:** 2025-09-25

**Authors:** Militsa Pacheva, Daniel Brugger, Barbara Riond, Peter Hendrik Kook

**Affiliations:** 1 Clinic for Small Animal Internal Medicine, Vetsuisse Faculty, University of Zurich, 8057 Zurich, Switzerland; 2 Institute of Animal Nutrition and Dietetics, Vetsuisse Faculty, University of Zurich, 8057 Zurich, Switzerland; 3 Clinical Laboratory, Department for Clinical Diagnostics and Services, Vetsuisse Faculty, University of Zurich, 8057 Zurich, Switzerland

**Keywords:** cats, bile acids, corticosteroids, liver enzymes

## Abstract

While corticosteroids affect bile acid metabolism in humans and dogs, their impact on serum bile acids in cats is unknown. Moreover, the early effects of corticosteroids on liver enzyme activity in cats are not well understood. We prospectively studied seven healthy cats (4–7 years) treated with oral prednisolone (1.1–1.5 mg/kg, median 1.28 mg/kg PO daily) for 7 days. Serum bile acids and liver enzyme activities (ALT, AST, ALP, GGT) were measured before treatment (day 0) and at days 2, 3, 8, 10, and 14. Statistical analysis used the Friedman test with post hoc comparisons to baseline. At baseline, bile acids were within reference interval (1–6.5 μmol/L) (median 2.1 μmol/L, range 1–3.2) and increased significantly during treatment with prednisolone to a median of 5.3 μmol/L (range 1.8–8.7) at d8, but remained below clinically abnormal levels. In contrast, AST, ALT, and ALP activities decreased significantly during prednisolone administration. GGT activities were undetectable at all times. We conclude that, although prednisolone significantly affected bile acids, the effect is likely of limited clinical relevance, as increases were minimal and remained below thresholds considered abnormal. Unexpectedly, transaminase and alkaline phosphatase activities decreased in the first days after administration. The reasons for this remain unclear.

## Simple Summary

Corticosteroids like prednisolone are commonly used in veterinary medicine, including in cats. To date, the effects of corticosteroids on bile acid levels in cats have not been studied at all, and early changes in liver enzyme activities within the first days after treatment initiation have also not been investigated. In our study, we gave seven healthy cats a daily dose of oral prednisolone for one week and monitored their blood over two weeks to track changes in bile acids and liver enzymes (ALT, AST, ALP, and GGT). We found that bile acid levels increased slightly during treatment but stayed within or just above the normal range. Although this change was statistically significant, it is likely not clinically relevant. Interestingly, instead of increasing, liver enzyme activities actually decreased in the early days of treatment. GGT remained undetectable throughout. Our findings suggest that short-term prednisolone use causes a small, likely unimportant increase in bile acids in cats. The decrease in liver enzymes, which goes against common expectations, highlights the need for further research into the early effects of corticosteroids on the feline liver.

## 1. Introduction

Liver disease is common in cats, and the majority of these cases is due to inflammatory liver disorders [1,2]. Assessment of hepatic health in cats typically includes measurements of transaminase activities (ALT and AST), alkaline phosphatase (ALP) and gamma-glutamyl transferase (GGT) activities, as well as bilirubin, cholesterol, and bile acids. These parameters are utilized not only in the diagnosis of liver disease but also for ongoing monitoring of disease progression and therapeutic response. Various hepatic disorders affect bile acid uptake and clearance in hepatocytes, making serum bile acids one of the most sensitive laboratory markers for evaluating liver function in small animal medicine [3].

Corticosteroids are the most commonly used treatment for non-infectious inflammatory liver disease in cats [4,5]. While some effects of corticosteroids on ALP, ALT, AST, and GGT activities have been studied in felines [6,7,8], it has never been examined to what extent corticosteroids influence bile acid concentrations in this species.

Bile acids are organic acids synthesized from cholesterol by hepatocytes and excreted into the bile. After their synthesis in the liver, they are conjugated with glycine or taurine to increase solubility, secreted into the biliary system and released into the duodenum, where they emulsify dietary lipids and facilitate micelle formation for fat digestion and absorption; the majority are actively reabsorbed in the terminal ileum and returned to the liver via the portal vein, completing the enterohepatic circulation, while a smaller fraction escapes to the colon where bacterial metabolism generates secondary bile acids. Their synthesis, secretion, and intestinal reabsorption are influenced by various factors, such as intestinal malabsorption, bile duct obstruction, hepatic failure, portosystemic shunting, and hormones, including glucocorticoids and thyroxin.

Studies with mice and rats have shown that glucocorticoids suppress bile acid synthesis by downregulating the Cyp7a1 (cholesterol 7α-hydroxylase), which is an important rate-limiting enzyme for bile acid production [9,10]. At the same time, they increase the expression of bile acid transporters in both the intestine and the liver, thereby enhancing bile acid absorption and hepatic uptake. These effects are thought to contribute to the elevation of the circulating bile acid concentration.

Only two studies report on the influence of corticosteroids on serum bile acids in dogs. These showed no clinically relevant increase in serum bile acids in dogs treated with anti-inflammatory or immunosuppressive dosages of corticosteroids [11,12].

With this study, we aimed to fill the gap and examine the effects of prednisolone on serum bile acids in healthy cats. A secondary aim was to investigate the effects of prednisolone on routine hepatic enzyme activities (ALT, AST, GGT, ALP), as existing data are limited, particularly regarding the early changes during the first days of treatment.

## 2. Materials and Methods

The results presented in this study are based on data collected as part of a study previously conducted by the authors, which examined the effects of prednisolone on serum lipase activity and pancreatic lipase immunoreactivity concentration in cats [13].

Seven European mixed-breed cats from a research colony of the Institute of Animal Nutrition and Dietetics of the Vetsuisse Faculty, University of Zurich, Switzerland were enrolled in the study. The cats were housed in an indoor-outdoor colony facility under the daily supervision of certified technicians and veterinarians. All seven cats were clinically healthy at the time of enrollment and had stable body weight and no health issues for the 12 months preceding the study. Inclusion criteria required a normal clinical examination, and normal results of a complete blood count, and serum biochemistry profile before enrollment. None of the cats had participated in any experiments within the past 18 months, and prior involvement was limited to feeding trials. All cats were maintained on their usual diet (Royal Canin Expert Adult Cat), provided ad libitum [13].

This was a prospective longitudinal observational study. Prednisolone (Hedylon, 5 mg tablets, Dr. E. Graeub, Berne, Switzerland) at a median dose of 1.3 mg/kg (range, 1.1–1.5 mg/kg) was administered orally in the morning for 7 consecutive days. The results from day (d) 1 are also baseline values, as blood samples were collected immediately before prednisolone administration. Blood was collected further on days 2, 3, 8, 10, and 14. Blood samples were stored at room temperature and brought to the laboratory within 2 h after collection, and all measurements were performed immediately upon arrival. Throughout the study period, all cats received daily physical examinations by two clinicians (an internal medicine resident and a board-certified internist) and were weighed each day. Discontinuation criteria included body weight loss > 5%, inappetence, vomiting, or diarrhea lasting longer than one day [13].

Measurement of serum bile acid concentrations (Diazyme Total Bile Acid Assay, Diazyme Laboratories, Inc., Poway, CA 92064, USA), alkaline phosphatase activity (ALP), aspartate aminotransferase activity, alanine transaminase activity (ALT) (all enzymes Roche on Cobas Integra, Roche Diagnostics), total protein concentration, and albumin concentration was performed with a Cobas C501 module (Roche Diagnostics, 6343 Rotkreuz, Switzerland).

The minimum sample size for the present study was estimated with G*Power (version 3.1.9.7, Heinrich Heine University, Düsseldorf, Germany) [14]. The basis for the cat number used was a calculation from a previous study, where the possible effect of prednisolone on lipase levels in cats was investigated [13]. The normality of data was assessed using the Shapiro–Wilk test. As not all data sets were normally distributed, descriptive data were presented as the median and range. A Friedman test was performed to compare serum bile acid concentrations and enzyme activities over time, followed by a post hoc Dunn’s test to compare all laboratory values at multiple time points with baseline (d0). When the Friedman test with post hoc analysis identified significant changes over time compared to baseline, median values are reported descriptively, as the test is based on ranks.

A commercial statistical analysis program (Prism 6; GraphPad, Boston, MA, USA) was used for statistical analyses. For all analyses, a *p* <0.05 was considered statistically significant.

The study was conducted in accordance with guidelines established by the Animal Welfare Act of Switzerland (No. ZH195/2021) and was approved by the Cantonal Veterinary Office of Zurich.

## 3. Results

### 3.1. Bile Acid Concentrations

**Figure 1:**
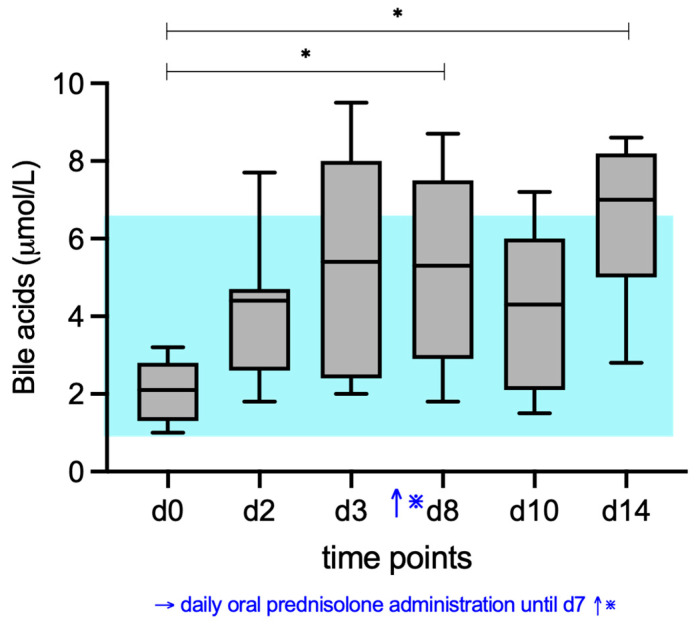
Median serum bile acid concentrations of the cats given prednisolone. Prednisolone was given daily from d1 to d7. The blue shaded area represents the reference interval. Friedman test with post hoc comparisons; * indicates *p* < 0.05.

At d0, all 7 cats had serum bile acid concentrations well within RI (1–6.5 µmol/L). Serum bile acid concentrations increased significantly compared to baseline (d0), with significant differences found between d0 (median 2.1 µmol/L) and d8 (median 5.3 µmol/L) (*p* = 0.0214), and d0 and d14 (median 7 µmol/L (*p* = 0.0084). Compared to the upper end of the RI (6.5 µmol/L), 1 cat at d2, 3 cats at d3, 2 cats at d8, 1 cat at d10, and 3 cats at d14 had serum bile acid concentrations over the upper limit of the RI (Figure 1).

### 3.2. ALP, AST, ALT, and GGT Activity

**Figure 2:**
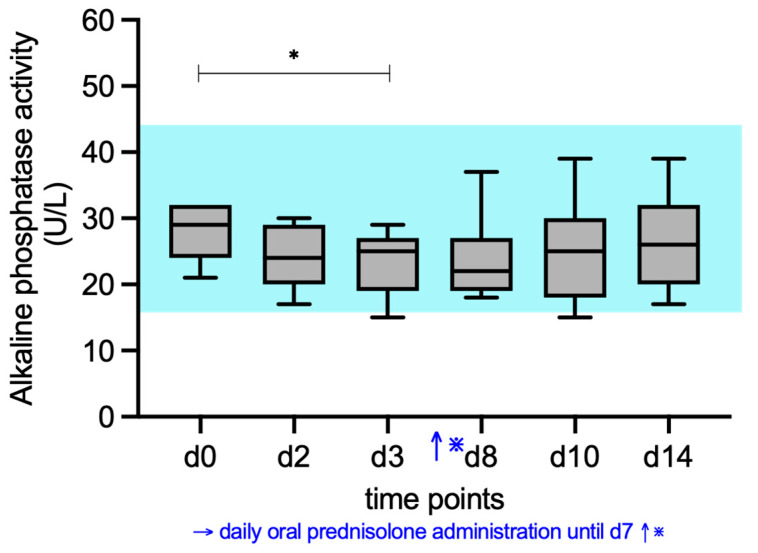
Median serum ALP activity of the cats given prednisolone. Prednisolone was given daily from day d1 to d7. The blue shaded area represents the reference interval. Friedman test with post hoc comparisons; * indicates *p* < 0.05.

ALP activities (RI, 16–43 U/L) differed significantly during prednisolone treatment compared to d0 (Figure 2). Median alkaline phosphatase activity decreased significantly (*p* = 0.0332) at d3 (25 U/L) compared to d0 (29 U/L). All values except one at d3 (15 U/L) remained within RI.

**Figure 3:**
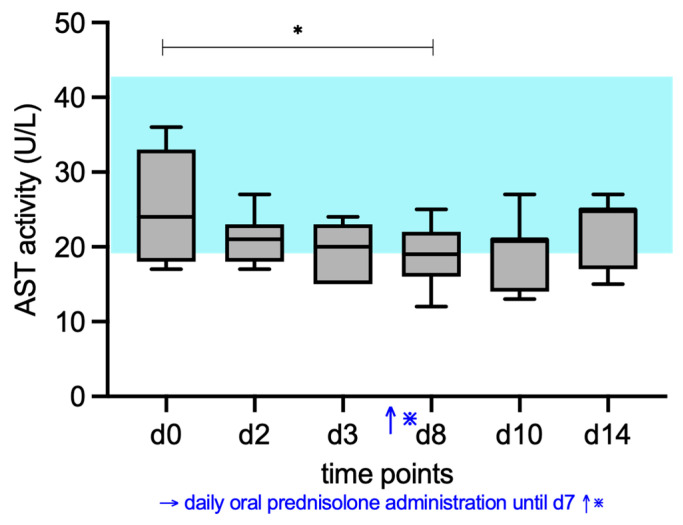
Median serum AST activity of the cats given prednisolone. Prednisolone was given daily from day d1 to d7. The blue shaded area represents the reference interval. Friedman test with post hoc comparisons; * indicates *p* < 0.05.

AST activities (RI, 19–44 U/L) decreased significantly during prednisolone treatment, and the post hoc test found a significant difference between d0 (median 24 U/L) and d8 (median 19 U/L) (*p* = 0.041) (Figure 3).

**Figure 4:**
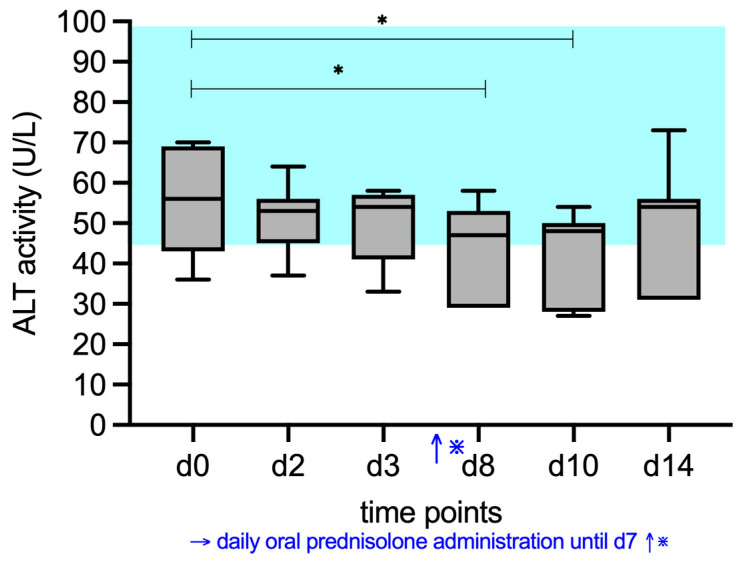
Median serum ALT activity of the cats given prednisolone. Prednisolone was given daily from day d1 to d7. The blue shaded area represents the reference interval. Friedman test with post hoc comparisons; * indicates *p* < 0.05.

ALT activities (RI, 43–98 U/L) also decreased significantly, and the post hoc test found a significant difference between d0 (median 56 U/L) and d8 (median 47 U/L) (*p* = 0.0411), and d0 and d10 (median 48 U/L) (*p* = 0.0214) (Figure 4).

GGT activities were not measurable at any time point in all 7 cats.

## 4. Discussion

Our findings indicate that prednisolone significantly influences serum bile acid concentrations; however, this effect appears to be of limited clinical relevance, as most values remained within the reference interval or exceeded it only marginally.

This is an important finding for clinicians, as prednisolone is frequently used in the treatment of inflammatory liver disease in cats. Moreover, inflammatory liver disease is often associated with chronic lymphoplasmacytic enteritis [15,16], a condition also commonly managed with prednisolone. Knowing that prednisolone does not cause a clinically relevant increase in serum bile acids in such cases helps veterinarians better assess the severity of liver disease and monitor its progression during treatment.

Our results are consistent with the findings of previous research in dogs. In a similar study of Pettersson et al., 10 dogs were treated with 1 mg/kg prednisolone for 10 days, and a significant increase in the serum bile acids was observed [11]. However, the increase was within the reference range and thus the clinical relevance also seems negligible. Another study with 8 dogs treated with immunosuppressive doses of 4 mg/kg prednisone showed again no relevant change in the serum bile acid concentrations [12]. In the same study, he concentration of hepatic bile acid in prednisone-treated dogs was also similar to that of the control group.

Experimental studies have shown that treatment with high doses of corticosteroids significantly increases serum bile acids in rats and mice [9,10]. This is believed to be due to increased enterohepatic recycling of bile acids, resulting in elevated plasma bile acid levels and reduced fecal bile excretion. However, it should be mentioned that the corticosteroid dosages used in these studies were noticeably higher (12 mg/kg/d and 20 mg/kg/d prednisolone) than standard doses used in cats with inflammatory disease, which could be a possible explanation for the different results.

Endogenous corticosteroids are also known to increase serum bile acids. Up to 30% of dogs with hyperadrenocorticism have increased pre- and postprandial bile acid concentrations [17,18,19]. It is assumed that this is the result of some degree of cholestasis, and mild increases are generally not considered to be clinically important. Similarly, serum bile acids were found to be increased in humans with hyperadrenocorticism. The proposed mechanism is that corticosteroids promote hepatic cholestasis by inhibiting the transcriptional activity of the Farnesoid X Receptor [20].

In this study, we additionally investigated the effect of prednisolone on standard liver enzymes, as prior studies did not capture the first days after initiation of corticosteroid therapy.

We found a slight decrease when evaluating the effect of prednisolone on ALP activity in cats. It is well established that corticosteroids do not induce a significant increase in ALP activity in cats, as they do in dogs [8,21,22]. Interestingly, in one study, ALP activity in 11 cats treated with a single injection of methylprednisolone decreased 3 to 6 days after injection, although this decrease was not statistically significant [6]. ALP activities increased significantly when re-assessed 2 to 3 weeks later, but the underlying mechanism is unclear. A slight decrease in ALP activity was also noted in the 2 cats treated with prednisolone in a study by Hoffmann et. al. and in 3 of 4 cats injected weekly for 4 weeks with methylprednisolone in another older study [22,23]. Our results confirm the trend observed by these previous studies, namely, a decrease in ALP activity during the first days after corticosteroid administration. A reduction in ALP levels has also been reported in humans undergoing corticosteroid treatment [24,25]. This is believed to be due to reduced osteoblast activity and bone formation. Although a decreased alkaline phosphatase activity was an unexpected finding in our study, it currently has no clinical relevance.

A transient decrease in AST and ALT activity was also observed after 7 days of prednisolone administration, which appeared to be reversible. The underlying mechanism remains unclear. A dilutional effect is unlikely, given that both albumin and total protein concentrations increased during treatment (Supplementary Figures S1 and S2). Prednisolone may stabilize cell membranes and reduce the cellular release of enzymes under non-pathological conditions. Alternatively, gene expression profiles might be altered under prednisolone, which could result in a reduction in the AST and ALT enzymes themselves.

GGT is an enzyme that regulates amino acid transport across cell membranes and is critical for the cellular redox pathway. GGT is most markedly increased in cats with necroinflammatory liver diseases, major bile duct obstruction, or inflammatory intrahepatic cholestasis. Some studies have shown that corticosteroids may stimulate GGT synthesis in dogs [26,27]. To date, the effects of corticosteroids on GGT activity in cats have only been described in a single study, in which GGT levels were assessed once after 56 days of immunosuppressive corticosteroid treatment [7]. Similarly to the findings of that longer-term study, our results now show no effect of prednisolone on GGT activity during the initial days of corticosteroid administration.

Our study also had some limitations. An a priori power analysis was performed to assess sample size, but it was concerned with the statistical power with regard to possible effects of prednisolone on lipase activity, rather than on bile acid concentration [13]. Although the limited sample size is considered a major limitation, the findings of the present study are consistent across all cats in this study and align with previous research, conducted with comparably small cohorts. Nonetheless, our data should be considered preliminary, and subsequent studies with larger sample sizes are warranted to confirm these findings.

A limitation of this study is that blood samples were collected from cats in a non-fasted state, as they were fed ad libitum. However, this was consistent across all sampling time points, and a clear temporal trend was still observed. In clinical practice, cats are often not fasted before blood sampling, and therefore, our results likely reflect real-world conditions well.

While measuring both fasting and postprandial samples is preferred due to their increased sensitivity in detecting hepatic dysfunction, this is often not practical in daily clinical practice. At the same time, postprandial bile acids have been shown to have the highest sensitivity of any single test in cats with hepatobiliary disease [28]. In general, bile acid concentrations greater than 25 μmol/L are considered to be suggestive of hepatobiliary disease in cats, a threshold that applies to preprandial (fasting), postprandial, and random (unrelated to eating) samples [3]. Most veterinary reference laboratories, like ours, use cutoff values for postprandial or random bile acids in cats between 15 and 30 μmol/L. Since none of the measurements in this study exceeded even 10 μmol/L, we conclude that although anti-inflammatory dosages of prednisolone significantly increased serum bile acid concentrations in healthy cats, this increase is not clinically significant. Further research is warranted to determine whether prednisolone has an effect on strictly preprandial and postprandial bile acid levels in cats.

## 5. Conclusions

Short-term administration of prednisolone did not have a clinically relevant effect on serum bile acids and liver enzymes in a small cohort of cats. Further research with a larger study group may be needed to confirm these findings.

## Data Availability

The original contributions presented in this study are included in the article/supplementary material. Further inquiries can be directed to the corresponding author(s).

## Supplementary Materials

The following supporting information can be downloaded at: https://www.mdpi.com/article/10.3390/vetsci12100933/s1, Figure S1: Serum albumin concentrations; Figure S2: Serum protein concentrations.

## Abbreviations

The following abbreviations are used in this manuscript:

ALT	Alanine aminotransferase
AST	Aspartate aminotransferase
ALP	Alkaline phosphatase
GGP	γ-glutamyltransferase
RI	Reference interval
*p*	*p*-value
CBC	Complete blood count
