# Supplementary material for: Influence of Prednisolone Treatment on Serum Bile Acid Concentrations in Cats
Source: Vet Sci. 2025 Sep 25;12(10):933. doi: 10.3390/vetsci12100933 (PMC12567590; doi:10.3390/vetsci12100933)
Supplement: Supplementary file 1 [file vetsci-12-00933-s001.zip › vetsci-3882371-supplementary.pdf]

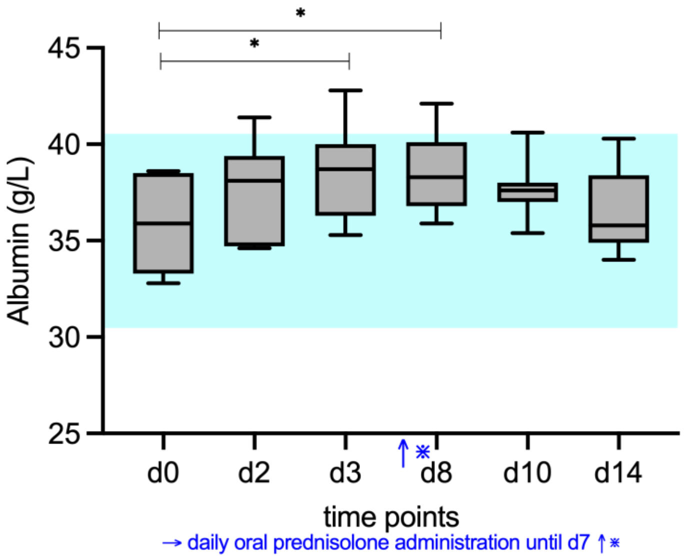

Figure S1: Median serum albumin concentration of the cats given prednisolone. Prednisolone was given daily from day d1 to d7. The blue shaded area symbolizes the reference interval. Friedman test with post hoc comparisons; * indicates $p < 0.05$.

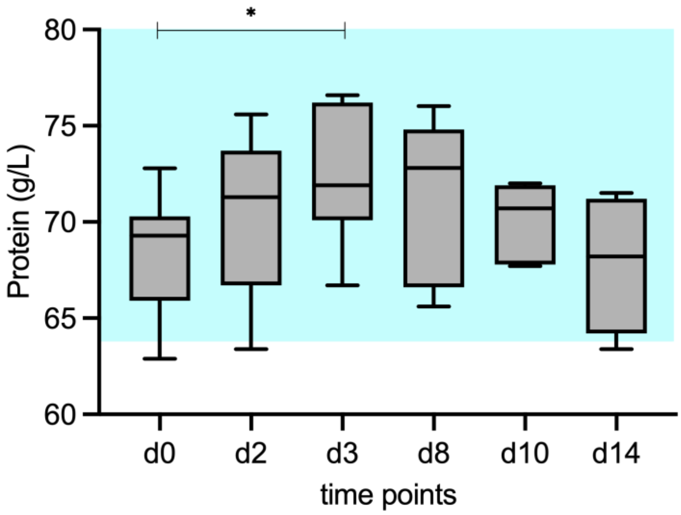

Figure S2: Median serum protein concentration of the cats given prednisolone. Prednisolone was given daily from day d1 to d7. The blue shaded area symbolizes the reference interval. Friedman test with post hoc comparisons; * indicates $p < 0.05$.
